# Retraction Note: Non-coding RNAome of RPE cells under oxidative stress suggests unknown regulative aspects of Retinitis pigmentosa etiopathogenesis

**DOI:** 10.1038/s41598-019-50646-7

**Published:** 2019-09-25

**Authors:** Luigi Donato, Concetta Scimone, Carmela Rinaldi, Rosalia D’Angelo, Antonina Sidoti

**Affiliations:** 1 Department of Biomedical and Dental Sciences and Morphofunctional Imaging, Division of Medical Biotechnologies and Preventive Medicine, University of Messina, Messina, Italy; 2 Department of Cutting-Edge Medicine and Therapies, Biomolecular Strategies and Neuroscience, Section of Applied Neuroscience, Molecular Genetics and Predictive Medicine, I.E.ME.S.T, Palermo, Italy; 3 Department of Chemical, Biological, Pharmaceutical and Environmental Sciences, University of Messina, Messina, Italy

Retraction of: *Scientific Reports* 10.1038/s41598-018-35086-z, published online 09 November 2018

Authors retract this Article.

In the study the Authors used an RNA-seq dataset that was not generated in their laboratory and was not publicly available for re-use.

All Authors agree to the retraction.

